# The Biochemical Assessment of Mitochondrial Respiratory Chain Disorders

**DOI:** 10.3390/ijms23137487

**Published:** 2022-07-05

**Authors:** Nadia Turton, Neve Cufflin, Mollie Dewsbury, Olivia Fitzpatrick, Rahida Islam, Lowidka Linares Watler, Cara McPartland, Sophie Whitelaw, Caitlin Connor, Charlotte Morris, Jason Fang, Ollie Gartland, Liv Holt, Iain P. Hargreaves

**Affiliations:** School of Pharmacy and Biomolecular Sciences, Liverpool John Moores University, Liverpool L3 3AF, UK; n.m.turton@2020.ljmu.ac.uk (N.T.); n.m.cufflin@2019.ljmu.ac.uk (N.C.); m.dewsbury@2019.ljmu.ac.uk (M.D.); o.a.fitzpatrick@2019.ljmu.ac.uk (O.F.); r.b.islam@2019.ljmu.ac.uk (R.I.); l.s.linareswatler@2019.ljmu.ac.uk (L.L.W.); c.m.mcpartland@2019.ljmu.ac.uk (C.M.); s.whitelaw@2019.ljmu.ac.uk (S.W.); c.e.connor@2018.ljmu.ac.uk (C.C.); c.l.morris@2019.ljmu.ac.uk (C.M.); g.f.fang@2018.ljmu.ac.uk (J.F.); o.j.gartland@2019.ljmu.ac.uk (O.G.); o.c.holt@2018.ljmu.ac.uk (L.H.)

**Keywords:** mitochondrial respiratory chain, lactate, pyruvate, organic acids, spectrophotometric enzyme assay, polarography, FGF21, biomarkers, amino acids

## Abstract

Mitochondrial respiratory chain (MRC) disorders are a complex group of diseases whose diagnosis requires a multidisciplinary approach in which the biochemical investigations play an important role. Initial investigations include metabolite analysis in both blood and urine and the measurement of lactate, pyruvate and amino acid levels, as well as urine organic acids. Recently, hormone-like cytokines, such as fibroblast growth factor-21 (FGF-21), have also been used as a means of assessing evidence of MRC dysfunction, although work is still required to confirm their diagnostic utility and reliability. The assessment of evidence of oxidative stress may also be an important parameter to consider in the diagnosis of MRC function in view of its association with mitochondrial dysfunction. At present, due to the lack of reliable biomarkers available for assessing evidence of MRC dysfunction, the spectrophotometric determination of MRC enzyme activities in skeletal muscle or tissue from the disease-presenting organ is considered the ‘Gold Standard’ biochemical method to provide evidence of MRC dysfunction. The purpose of this review is to outline a number of biochemical methods that may provide diagnostic evidence of MRC dysfunction in patients.

## 1. Introduction

**Figure 1:**
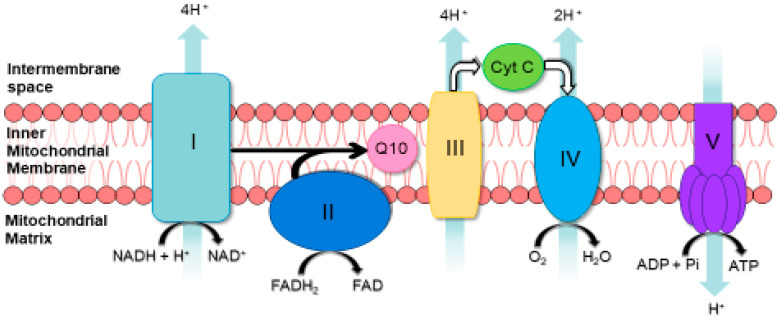
Diagram of the mitochondrial respiratory chain (MRC) and complex V illustrating proton (H+) movement during oxidative phosphorylation. Protons as pumping into the inner mitochondrial space occurs at complexes I, III and IV and the protons pass back into the matrix at complex V. This is known as the `proton circuit` and is illustrated by the arrows in the figure. Q10: Coenzyme Q_10_. Cyt C: Cytochrome c.

The mitochondrial respiratory chain (MRC; Figure 1) is composed of four enzyme complexes: complex I (NADH: ubiquinone reductase; EC 1.6.5.3); complex II (succinate: ubiquinone reductase; EC 1.3.5.1); complex III (ubiquinol: cytochrome c reductase; EC 1.10.2.2) and complex IV (cytochrome c oxidase; EC 1.9.3.1). It also includes two electron carriers, coenzyme Q10 (CoQ10) and cytochrome c, and is located in the inner mitochondrial membrane [1,2]. The MRC, together with complex V (ATP synthase; EC 3.6.3.14), synthesizes ATP by the process of oxidative phosphorylation [1,2]. MRC disorders represent the most common group of metabolic diseases, affecting approximately 1 in 5000 individuals [3]. In the UK, the incidence of MRC disorders has been estimated to be as high as 1 in 4300 [4]. Diagnosing evidence of MRC dysfunction is often challenging and fraught with difficulties in view of the extremely heterogeneous clinical presentation of these diseases. In a small proportion of patients with recognized clinical phenotypes, it may be possible to utilize genetic studies to achieve a diagnosis [5]. However, in view of the poor genotype–phenotype relationship of MRC disorders, an accurate diagnosis generally requires a multidisciplinary approach involving clinical, genetic, histological and biochemical investigations [5,6]. In biochemical investigations, metabolite analysis in blood and urine is usually performed prior to enzymatic studies in muscle biopsy [7]. The metabolite analysis includes the assessment of plasma lactate, pyruvate and amino acids together with the urine organic acid profile [7]. Although these initial investigations can provide an indication of MRC dysfunction, they can lack sensitivity and specificity [7]. The diagnosis of MRC disorders is impeded by the limited number of surrogates or biomarkers of MRC dysfunction, although the hormone-like cytokines, fibroblast growth factor-21 (FGF-21) and growth differentiation factor-15 (GDF-15) may have some utility as potential biomarkers [8,9]. The assessment of markers of oxidative stress, such as reduced glutathione (GSH), together with the cellular status of molecules that may influence MRC function, such as coenzyme Q10 (CoQ10) [10], may also provide important information on the origin of the MRC dysfunction. In addition, the assessment of the cerebral spinal fluid (CSF) status of the folate species, 5-methyltetrahydrofolate (5MHTF), may also be of value, since a deficit in the level of this molecule has been associated with MRC dysfunction [11]. At present, however, the spectrophotometric determination of MRC enzyme activities in a skeletal muscle biopsy or tissue from the disease-presenting organ is still considered the most reliable biochemical method for determining evidence of MRC dysfunction in patients [7]. The following review paper will outline the current biochemical methods utilised in the diagnosis of MRC disorders, as well as highlight potential biomarkers for this determination.

## 2. Lactate and Pyruvate

MRC disorders can be detected via blood serum, urine and CSF analysis, with increased lactate levels being a principal biochemical marker for mitochondrial abnormality [12]. An elevation in blood lactate is resultative of the impaired utilisation of pyruvate by the mitochondria during aerobic respiration. Under aerobic conditions, pyruvate is converted into acetyl CoA by pyruvate dehydrogenase, which may then enter Krebs cycle and be completely oxidised to CO_2_ and H_2_O [13]. However, when mitochondria are impaired, the resultant inability to fully oxidise pyruvate to drive ATP production causes pyruvate to be reduced into lactate. The reduction of pyruvate by NADH to produce lactate is catalysed by the lactate dehydrogenase enzyme. Whole blood samples, as well as serum/plasma and CSF samples with lactate levels >2.1 mM, provide indicative evidence of mitochondrial dysfunction, but this does not offer a specific diagnosis of MRC impairment. It is possible that raised blood lactate levels in a patient may be due to other medical issues such as cardiac dysfunction, which causes elevated blood lactate levels as a result of the heart’s inability to deliver oxygen-rich blood to highly metabolically active tissues [14]. Furthermore, an elevated plasma lactate level may also result from a patient struggling or from the prolonged use of a tourniquet during the collection of the blood sample [3,14]. A suggested strategy to resolve this problem is to collect the sample following 30 min placement of an intravenous catheter [3]. Poor nutritional status, such as a vitamin B1 (thiamine) deficiency, may also result in an elevated plasma lactate and/or pyruvate level since thiamine is an essential cofactor for pyruvate dehydrogenase activity [3,14]. Additionally, increases in CSF lactate levels can be a consequence of central nervous system (CNS) infections, seizures, inflammation or malignancies, but this is also a predominant characteristic in children with MRC disorders [15]. Furthermore, elevations in blood and/or CSF lactate may not always be present in patients with mitochondrial dysfunction [16]; thus, if a patient is found to have a lactate level within the reference range, this does not exclude the possibility of an underlying MRC disorder. When oxidative phosphorylation is impaired, the cytosolic NADH:NAD ratio is typically, but not exclusively, raised, which, in turn, reflects the molar ratio of lactate to pyruvate (L:P) [6]. Hence, an L:P value > 25, in conjunction with an increase in plasma lactate concentration, is a good, although not exclusive, indicator of MRC dysfunction [17]. Pyruvate is quite unstable, however, requiring the blood sample to be immediately transferred into a tube containing 8% perchlorate prior to analysis [3]. Blood and/or CSF pyruvate levels may also be elevated in patients with defects in pyruvate metabolism, such as pyruvate dehydrogenase deficiency, pyruvate carboxylase deficiency or biotinidase deficiency [3,14,17]. An elevated blood alanine concentration is also an indicator of accumulating pyruvate and is commonly observed in MRC disorders, as well as pyruvate dehydrogenase deficiency, pyruvate carboxylase deficiency and urea cycle disorders [18]. Due to their diagnostic limitations, an elevated plasma/CSF lactate level and/or L:P ratio is not sufficient to provide diagnostic evidence of an underlying MRC defect, although they may provide an indication of a possible perturbation in oxidative phosphorylation, which can then be confirmed/refuted with further biochemical, genetic or histological investigations [3,6,17,18].

## 3. Amino Acids

Elevated levels of amino acids, such as alanine, may be found with an amino acid analysis of the plasma and/or CSF of patients with MRC dysfunction. Alanine is a product of the transamination of pyruvate by the enzyme alanine aminotransferase [19] and is involved in the biosynthesis of proteins. Despite increased levels of alanine being reflective of a cellular accumulation of pyruvate and indicating evidence of MRC dysfunction, there are other possible explanations for this amino acid concentration being elevated. Patients with a deficiency in the enzyme pyruvate dehydrogenase (PDH) have shown a significantly larger increase in plasma alanine levels compared to those with mitochondrial disease [20]. Mitochondrial disease criteria (MDC) scoring systems, such as the modified Walker criteria [21], define abnormal metabolic studies, such as high alanine levels, as a minor criterion of mitochondrial dysfunction. According to the Nijmegan protocol, a plasma alanine level of >450 mM in patients is used to help determine the likelihood of mitochondrial dysfunction [21]. However, an increase in serum alanine concentrations has been identified in other conditions, such as sepsis, hyperinsulinism and chronic thiamine deficiency [22].

It is recommended that, when assessing evidence of hyperalaninaemia in patients, a plasma or CSF sample should be taken from a fasting patient. This is due to the ability of dietary intake to influence circulatory amino acid levels [6]. A study by Carayol et al. (2015) [20] found that the overall reproducibility of serum metabolite samples (including amino acids) was higher in fasting compared to non-fasting samples. Importantly, during a relapse in symptoms, there would be an elevated level of plasma alanine; thus, if a ‘normal’ plasma alanine level presents within an individual, this does not exclude an underlying mitochondrial dysfunction [6]. However, according to literature presented by Parikh et al., 2015 [16], the exact specificity and sensitivity of alanine elevations in patients with primary mitochondrial dysfunction are still to be elucidated. Clarke et al. (2013) [18] established a retrospective cohort study that suggested individuals who present with a raised alanine status alongside MRC dysfunction may also have raised branch chain amino acid (BCAA: leucine, isoleucine, valine) levels together with increased ratios of BCAAs to glutamate. It can therefore be assumed that the accumulation of BCAAs and alanine in patients with MRC dysfunction possibly reflects a rise in glutamate-linked transamination reactions, attributable to an impairment of NAD^+^-dependent keto-acid oxidation as a result of a diminution in the availability of this cofactor [18]. It is interesting to note that individuals presenting with PDH deficiency were not found to present with elevated plasma BCAA levels relative or absolutely to glutamate [19].

Research conducted by Dimou and colleagues in 2022 [21] provides further information about the relationship between MRC dysfunction and the increased circulatory levels of BCAAs. There are similarities between the present study and those described by Clark et al., 2013 [18], including how the accumulation of BCAAs—more specifically, leucine—inhibits the function of the enzymes (α-ketoglutarate dehydrogenase, pyruvate dehydrogenase and those of the MRC) and, in turn, provides further scientific evidence and support to older studies. The focus of recent literature has shifted towards BCAA homeostasis and how it holds great importance for brain function and its correlation with severe neurological diseases [21]. Therefore, a greater understanding of the mechanisms connecting the central nervous system to increasing levels of BCAA may aid novel therapeutic approaches to mitochondrial disorders. The assessment of urinary amino acid status is a useful method to assess for evidence of renal tubulopathy in individuals with MRC dysfunction, with generalised aminoaciduria occasionally reported [22]. This is consistent with a more recent study conducted by Shatla et al., 2014 [23], further supporting the use of urinary and plasma amino acids within the diagnosis of mitochondrial disease.

## 4. Urine Organic Acids

Organic acids are a family of compounds that are by-products and intermediates in many metabolic pathways associated with protein, carbohydrate and fat metabolism such as glycolysis, fatty acid oxidation, citric acid cycle, ketone metabolites and cofactors. They are also markers of detoxification and are present in urine, plasma and CSF [6,24]. Although all three matrixes can be used for organic acid analysis, due to its extraction efficiency, urine is the tissue of choice for this analysis [6]. Despite the fact that it is rare for a plasma or CSF sample to provide more diagnostic information than a urine sample, the assessment of organic acids in CSF for specific disorders has been suggested, such as in patients with suspected disorders of GABA metabolism [6].

Numerous organic acids are present in the urine of patients who are reported to be clinically normal, although the actual concentration range for each of these acids can differ greatly, which may limit their diagnostic utility [24]. In a retrospective cohort study by Alban and colleagues (2017), an abnormal organic acid profile was reported in 82% of the patients with a muscle MRC enzyme deficiency [25]. Patients with isolated MRC complex I and II deficiencies were found to have an elevated urine lactate level, and patients with isolated MRC complex IV and V deficiencies were found to have an increased urinary level of 3-methylglutaconic acid, which is an intermediate in mitochondrial metabolism of leucine, a branch chain amino acid [25]. Another common finding in the urine organic acid profile of these patients is the presence of elevated levels of dicarboxylic acids [6,26]. Dicarboxylic acids arise as the result of the ω-oxidation of fatty acids, which occurs in the smooth endoplasmic reticulum. The increase in urine dicarboxylic acids levels is thought to occur due to impaired fatty acid ω-oxidation, which can result as consequence of a defect in MRC function [6,26]. Although the analysis of urine organic acids is highly important in the diagnosis of MRC dysfunction, there are some limitations associated with this diagnosis; for example, a urine organic acid analysis of a patient with MRC dysfunction during a period of clinical stability may not reveal any evidence of a metabolic abnormality [27,28]. Furthermore, an abnormal urine organic profile can occur in a patient suffering from poor perfusion or dehydration at the time of sample collection [6,27,28]. Renal immaturity can result in the presence of TCA cycle intermediates in the urine of the patient and, therefore, an abnormal urine organic acid profile in a patient less than one year of age should be interpreted with extreme caution [6,28].

## 5. Other Metabolites

An elevated level of plasma creatine has been suggested as a potential biomarker of MRC dysfunction [29]. Creatine kinase phosphorylates creatine, converting it into creatine phosphate, which serves as a rapid mobilizable energy store that is present in skeletal muscle, liver and brain, where it is used as a recycling mechanism for ATP generation [29]. However, impaired MRC function may result in a perturbation of creatine synthesis and utilization, resulting in creatine being released into the bloodstream, where it may have utility as an indicator of MRC dysfunction [29]. However, in view of the number of parameters that may influence circulatory creatine levels, its value as a diagnostic marker requires further investigation [28]. Moreover, a study by Maresca et al. (2020) [30] investigated creatine serum levels in patients with various types of mitochondrial diseases and found that creatine levels were increased in patients with MRC dysfunction with a mean level of 12.11 μmol/L compared to 6.92 μmol/L in the control group. However, only patients with severe MRC dysfunction were found to have increased serum creatine levels. In addition, serum creatine levels were not able to differentiate between specific clinical phenotypes, or to distinguish whether the MRC dysfunction was caused by mutations in either mtDNA or nuclear DNA [30].

Patients with MRC dysfunction have been reported to present with a deficiency in plasma-free carnitine status, which, in some cases, may accompany an elevated level acyl-carnitine species [31]. Nevertheless, an abnormal carnitine/acyl carnitine profile may not be present in all patients [31]. The aberrant carnitine/acyl-carnitine profile possibly reflects the secondary impairments of fatty acid β-oxidation as the result of MRC dysfunction. Moreover, evaluating the diagnostic value of plasma carnitine/acyl-carnitine assessment becomes questionable when considering the limited background literature available to support its utility in identifying patients with MRC dysfunction [31].

## 6. Spectrophotometric Assessment of MRC Enzyme Activities

Currently, the spectrophotometric assay of MRC enzyme activities appears to be the most reliable biochemical method for determining evidence of MRC dysfunction in patients [3,6,7]. The use of detergents, or, more commonly, freezing and thawing, is required to permeabilize the inner mitochondrial membrane to ensure access to the required substrates utilised for determining MRC enzyme activities [32].

The spectrophotometric assay provides information on the maximal activities of the MRC enzymes and holds many benefits, such as being easy and readily reproducible. In contrast to this, these studies are not performed under physiological conditions, as pH and substrate concentration, as well as other assay conditions, are optimized in order to achieve the maximum activity measurements for the specific MRC enzymes [3,32]. Using spectrophotometric enzyme assays, the activities of MRC complexes I, II, III and IV can be determined (Table 1). In addition, the measurement of complexes II–III (succinate: cytochrome c reductase; EC. 1.3.5.1 + 1.10.2.2) and I–III (NADH: cytochrome c reductase; EC 1.6.5.3 + 1.10.2.2) provides evidence of a potential CoQ10 deficiency, since their activities are dependent on endogenous CoQ10 status [33]. MRC enzyme activities can be expressed either on a protein baseline, normalised to the total protein concentration of a tissue [34], or as a ratio to the activity of the mitochondrial marker enzyme citrate synthase (CS; EC 1.1.1.27), in order to take into account the mitochondrial enrichment of the tissue [33,34,35]. Expressing MRC enzyme activities as a ratio to CS activities takes into account age effects, removing the requirement for age-specific reference intervals [35]. Spectrophotometric enzyme assays are evaluated for reliability by the use of in-house quality controls, which can either be prepared from the remaining frozen skeletal muscle tissue stored with no evidence of an MRC disorder or from animal tissue, as no quality controls from these assays are available commercially [28]. Currently, there is no external quality assurance scheme that is available for the assessment of MRC enzyme activities and, therefore, specialist centres are not able to compare their results in order to reach a consensus on the appropriate diagnostic criteria required for the identification of MRC enzyme deficiencies in patients. Importantly, poor sample handling and/or storage of skeletal muscle has been reported to result in a reduction in the amount of mtDNA present within the tissue, which may also cause a concomitant loss of MRC enzyme activity. Therefore, it is important to handle the tissue correctly and according to the strict sampling instructions required for these determinations in order to avoid any artefactual loss of MRC function, which may result in a misdiagnosis [3,6,28].

## 7. Polarographic Assessment of MRC Function

**Figure 2:**
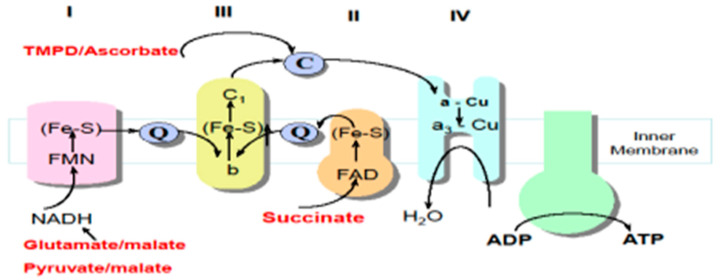
Substrates used in polarography to assess oxygen consumption rates illustrating their site of oxidation within the mitochondrial respiratory chain (MRC). C: cytochrome c, Q: CoQ10, TMPD: N,N,N′,N′-Tetramethyl-p-phenylenediamine.

Polarography measures the rate of oxygen consumption in cells with preserved mitochondrial integrity (intact and permeabilized cells or permeabilized tissue fragments), using a Clarke-type oxygen electrode in the presence of various substrates (Figure 2) [36]. The activity of the different regions of the MRC are probed by the various combinations of substrates used in this technique: glutamate + malate and/or pyruvate + malate generate NADH, which is oxidised at complex I; succinate generates FADH2, which is oxidised at complex II; and TMPD (N,N,N′,N′-Tetramethyl-p-phenylenediamine) + ascorbate, which donate electrons directly to cytochrome c (Figure 1) [6]. In the oxygen electrode chamber, ADP and inorganic phosphate (Pi, in respiratory buffer) are also required to enable the uptake of molecular oxygen (O_2_) by the mitochondria [32]. In the presence of ADP and Pi, the mitochondrial proton motive gradient is dissipated with the concomitant conversion of ADP and Pi into ATP by complex V with the resulting passage of electrons to O_2_, which is then reduced to water [32,37]. Respiration/oxygen consumption in the presence of ADP is termed state 3 [32]. The exhaustion of exogenous ADP, upon conversion to ATP, returns mitochondrial respiration to a resting state, state 4 [33]. The state 3: state 4 respiratory ratio, known as the respiratory control ratio (RCR), reveals information regarding the dependence of mitochondrial respiration–ATP synthesis coupling [38]. The collective use of various substrates in this method allows for an assessment of the integrated function of the MRC and the identification of potential enzyme defects [6,32,38]. MRC enzyme defects can then be confirmed spectrophotometrically [3,6,38]. Spectrophotometry and polarography are both important in the diagnosis of MRC disorders, giving complementary biochemical evidence [37]. The development of extremely sensitive, high-resolution respirometry apparatuses, such as the ‘Sea Horse’ and ‘Oroboros 2K’, present an opportunity to accurately analyse mitochondrial respiration in permeabilized cells, such as white blood cells or fibroblasts, and permeabilized muscle fibres from patients with suspected MRC disorders. These techniques diminish the traditional utilization of large, ‘mega’ biopsies (≥0.5 g of muscle tissue) for polarographic assessments of MRC function [38,39]. Furthermore, developments in microfluidics-based respirometry, utilising the VeroClear photopolymer, are comparable to high-resolution respirometry techniques [40]. This method measures O_2_ consumption rates in various biological samples (soluble enzyme systems, cell or organelle suspensions and adherent samples) in minute volumes without aerobic interference, beneficially yielding direct, uncompensated data and also removing high tissue demand associated with polarography [40]. However, the availability of these newly advanced protocols, together with the traditional Clarke-type electrodes, is variable within specialist centres, and, therefore, polarography is not considered an essential analytical technique in the diagnosis of MRC disorders [16].

## 8. CoQ10

The clinical determination of patient CoQ10 status is usually based upon measurements in plasma [33]. However, the levels of circulatory lipoproteins influence plasma CoQ10 status, as 58% of plasma CoQ10 is associated with the level of low-density lipoprotein fraction [41]. As well as this, it has been reported that ≤25% of total plasma CoQ10 may be of dietary origin [33]. Due to this, the assessment of endogenous CoQ10 status in patients may be more suitable in an alternative tissue, for example, skeletal muscle [33]. Evidence of CoQ10 in this tissue can be indicated initially by a reduction in the activity of MRC complexes I–III or II–III, and the residual homogenate can be utilised for the measurement of endogenous CoQ10 status. In this assessment, it should be noted that, in the skeletal muscle tissue of patients with MRC disorders, which are linked to mitochondrial proliferation, expressing skeletal muscle CoQ10 status to the total protein content of tissue could produce ‘false negative’ results [33,42]. Thus, to identify possible CoQ10 deficiencies in these patients, expressing tissue CoQ10 status to CS activity (marker of mitochondrial enrichment) may improve diagnostic yield [33]. When determining CoQ10 status, it is also critical to take into account mitochondrial abundance in tissue and cell samples, as approximately 50% of cellular CoQ10 status is present within this organelle. Other cell samples appropriate for CoQ10 assessment in patients with MRC disorders include blood mononuclear cells (BMNCs) and fibroblasts and, therefore, should be considered [33,43]. Patients with a primary CoQ10 deficiency (CoQ10 deficiency caused by a genetic defect in the CoQ10 biosynthetic pathway) constitute an important subgroup of mitochondrial patients where clinical improvement has been reported in response to supplementation with CoQ10 [33,43]. Although the determination of tissue CoQ10 status cannot distinguish between primary and secondary causes of CoQ10 deficiency (CoQ10 deficiencies not caused by a genetic defect in the CoQ10 biosynthetic pathway), radiolabelled incorporation studies in fibroblasts can enable discrimination between primary and secondary causes of CoQ10 deficiency [33].

The most conventional methods used to determine CoQ10 status in patients are either high-pressure liquid chromatography with electrochemical detection (HPLC-ED) or ultraviolet (HPLC-UV) [33]. It should be noted that, when assessing CoQ10 status by HPLC-UV, the lack of commercially available internal standards that are not influenced by endogenous/exogenous ubiquinones has made CoQ10 assessment a major challenge. However, Duncan et al. [43] have synthesized a non-physiologic internal standard (di-proxy CoQ10), which may be suitable for the determination of cellular CoQ10 status. Alternatively, methods, such as tandem mass spectrometry, have also been employed for this determination [33].

## 9. GSH

Patients with MRC disorders have also reported evidence of a deficit in the status of the antioxidant GSH [44]. This deficit is indicative of evidence of oxidative stress, which is prominent in the pathophysiology of many MRC disorders. However, since GSH synthesis is also ATP-dependant [44], the measurement of the ratio of GSH:GSSG (the oxidised disulphide form of GSH) has been suggested as a more appropriate surrogate for evaluating evidence of oxidative stress/cellular toxicity in patients [45]. In resting cells, the molar GSH: GSSG ratio exceeds 100:1, whilst in models of oxidative stress, this ratio has been observed at lower values of 10:1 and even 1:1 [46]. Sample handling can affect the levels of both GSH and GSSG, as GSH can oxidise into GSSG when conditions are not appropriately controlled [45]. Freeze clamping tissues with liquid nitrogen-cooled tongs and storage at −80 °C, or plasma and tissue sample acidification, can minimize autooxidation and degradation [47]. Measuring the endogenous GSH status of patients also allows for the therapeutic monitoring of treatments such as the vatiquinone, EPI-743, which is able to replenish patient GSH levels. This tripeptide is most often used to treat Friedreich’s ataxia (FA), as well as MRC disorders.

Skeletal muscle and leukocytes are the most reliable surrogates for determining patient GSH and GSSG levels [45]. However, levels have also been determined in whole blood, plasma, erythrocytes and urine, although biological samples such as plasma can be problematic due to the sensitivity required for quantification (1–10 µM) [47]. Many methods have been used for GSH:GSSG analysis, including fluorometer, spectrophotometric and bioluminescence assays, which are often applied to HPLC (HPLC-ED and HPLC-fluorescence detection (FD)). In addition to these methods, liquid chromatography–tandem mass spectrometry (LC-MS/MS) has also been used [45].

## 10. 5MTHF

A deficiency in cerebrospinal fluid (CSF) 5MTHF status has been reported in patients with many forms of mitochondrial disease, including Kearns-Sayre syndrome (KSS). The transport of 5MTHF across the blood CSF barrier (choroid plexus) happens via an active transport mechanism [48]. Thus, a deficit in 5MTHF status in the CSF is thought to reflect a deficiency in the availability of ATP. However, in KSS, adverse anatomical changes in the choroid plexus can also limit the movement of 5MTHF into the CSF [49]. A diminution in the CSF 5MTHF status of patients with MRC disorders may also be associated with the free radical-induced oxidative degradation of this folate species, as demonstrated by Aylett et al. [50], who showed a significant decrease in 5MTHF status in association with an increase in superoxide generation under in vitro conditions.

In order to determine the status of 5MTHF in the CSF, a lumbar punch must be performed after 4–6 h of fasting. CSF samples must be protected from light and frozen at −80 °C within 2 h of collection [51,52]. CSF 5MTHF status is usually determined by HPLC-FD, where the thawed CSF is directly injected into the HPLC [52]. However, 5MTHF can also be determined with liquid chromatography–tandem mass spectrometry (LC-MS/MS) [53]. A correlation between the CSF 5MTHF concentration and the total folate concentration in CSF has previously been identified, whereby a folate deficiency could be due to inadequate dietary intake (since 5MTHF and other folate compounds are part of the vitamin B family: vitamin B9). The estimation of 5MTHF from the total folate should only be used when there is limited access to specialised laboratories that measure 5MTHF [51].

In addition to determining CSF 5MTHF in mitochondrial disease patients, the level of ascorbic acid in the CSF should also be measured, as the antioxidant, ascorbic acid protects 5MTHF from ROS-induced degradation [50]. Thus, a deficit in the level of both 5MTHF and ascorbic acid in the CSF may be indicative that the loss of 5MTHF is due to an increased degradation of folate species by ROS instead of impaired active transport across the choroid plexus.

## 11. FGF-21/GF-15

The investigation of MRC disorders can be challenging due to struggles in diagnosis. However, there are reliable biomarkers present to assess evidence of MRC disorders that are more favourable, as they are less invasive—traditional methods include muscle biopsy and the spectrophotometric assessment of MRC enzyme activities [54] (Scholle et al., 2018). Studies have indicated that fibroblast growth factor 21 (FGF21) [55] and differentiation factor 15 (GDF15) [9] are reliable biomarkers of MRC dysfunction.

FGF21 is a metabolic hormone that is produced within the liver and is expressed in the pancreas and adipocytes [56]. The main role of FGF21 is to regulate glucose and lipid metabolism. In studies using obese mice and diabetic monkeys, FGF21 has shown a positive effect on many metabolic processes, such as blood glucose, lipid profile, insulin sensitivity and body weight, without mitogenic effects. Although this seems useful in treating obesity-related disorders, within humans, high FGF21 levels can indicate diseases, such as diabetes, obesity and renal dysfunction [55]. Therefore, the level of this cytokine in the blood may not be solely influenced by MRC dysfunction, limiting its diagnostic utility. However, it has been reported that, in mouse skeletal muscle, mitochondrial dysfunction induces FGF21 expression; thus, increased levels of the cytokine [57] may correlate with disease severity and progression in patients with muscle-presenting MRC deficiencies [8] In a subsequent study, FGF21 was found to be effective at discriminating patients with defects in mtDNA maintenance and single rearrangements from controls [55]. A study by Lehtonen et al., 2016 [58], indicated that serum-FGF21 can be used as a biomarker of muscle-manifesting mitochondrial maintenance and translational defects. However, a disadvantage of FGF-21 is that it may not be elevated in patients with non-muscle-presenting MRC disorders [59].

GDF15 is a similar biomarker to FGF21; however, GDF15 is more sensitive in detecting mitochondrial dysfunction affecting other organs [60]; thus, it is more sensitive but less specific. GDF15 presents with the same issues as FGF21: useful as a marker of mitochondrial myopathy but less specific in non-muscle-presenting MRC disorders [30]. Interestingly, the combined assessment of the serum levels of both FGF-21 and GDF-15 from adult patients with mitochondrial disease has not been found to improve the diagnostic value of the individual tests [61]. Intriguingly, a recent study by Riley et al., 2022 [62], showed that FGF21 performs better than GDF15 as a biomarker. The study used a healthy group of children without mitochondrial disease and compared them to those with MRC; results suggested that FGF21 outperforms GDF15 when discriminating between both groups. A study by Varhaug et al., 2021 [60], suggests the introduction of the biomarker neurofilament light-chain (NfL, a biomarker of neuro-axonal injury and neurodegeneration) due to its ability to identify mitochondrial patients presenting with CNS damage [63]. Furthermore, Varhaug et al., 2021, proposes using NfL in conjunction with FGF21, GDF15 and cell-free mitochondrial DNA, as this may narrow the choice of diagnostic test in cases of suspected mitochondrial disease [60]. Perhaps introducing more biomarkers to diagnose mitochondrial disease could produce specific results. Importantly, FGF21 is reportedly stable in whole blood or serum for up to three days at room temperature [55]. NfL serum and plasma samples can also remain stable for up to seven days at room temperature [64]. However, GDF15 only remains stable in serum, plasma and whole blood for at least 48 h [65].

## 12. Conclusions

At present, although there are a number of biochemical assays that can provide evidence of MRC dysfunction, the ‘Gold Standard’ investigation for this assessment is still considered to be the spectrophotometric determination of MRC enzyme activities in skeletal muscle or tissue from the disease-presenting organ, if accessible [7]. However, complex V activity cannot be accurately determined with spectrophotometric enzyme assays, and instead relies on blue native gel electrophoresis for its determination [66]. Furthermore, this method can be utilised for studying the super-complex formation of the enzymes of the MRCE, which may have important implications for disease pathophysiology, as indicated in the secondary mitochondrial disease, Barth syndrome [67,68]. Unfortunately, spectrophotometric enzymes, together with all currently available biochemical investigations, cannot distinguish between primary or secondary causes of MRC dysfunction, relying on concomitant genetic studies to elucidate the origin of the defect. However, with regard to primary and secondary CoQ10 deficiencies, radiolabelled studies in fibroblasts can be used to determine the origin of the deficiency [33]. Interestingly, although oxidative stress has been implicated as a contributory factor to the pathophysiology of MRC dysfunction, few, if any, specialist centres assess or monitor evidence of this parameter in patients [44]. Whilst the identification of evidence of oxidative stress may not be a specific indicator of MRC dysfunction, it may serve to alert the clinician of the requirement for antioxidant therapy, as well as to monitor the efficacy of this treatment. The assessment of leucocyte GSH levels may serve as the most accessible and reliable method of determining evidence of oxidative stress in patients, as an increase in the levels of this tripeptide have been associated with clinical improvement in patients [45,69].

There is a lot of interest in identifying appropriate biomarkers of MRC dysfunction in order to alleviate the need for an invasive muscle biopsy [7]. However, with limitations, the biomarkers FGF-21 and GDF-15 are currently available and have demonstrated some degree of clinical utility [55] for patients with muscle involvement. Moreover, the combined use of these biomarkers (either FGF-21 or GDF-15), together with NfL and cell-free mitochondrial DNA in serum, may improve the diagnostic yield for patients with suspected MRC dysfunction [60], although these biomarkers are indicators of tissue damage per se and may not specifically reflect MRC dysfunction. However, it is hoped that the employment of mass spectrometry omics investigations (metabolomics, proteomics and lipidomics) in animal models of MRC disease, or in tissue samples from patients with genetically defined MRC defects, will provide ‘signature molecules or metabolites’ that can be used in the future for the accurate diagnosis of patients with MRC dysfunction [70].

## Figures and Tables

**Table 1:** Details of spectrophotometric enzyme assays used to determine mitochondrial respiratory chain (MRC) enzymes and citrate synthase (CS) activities.

Enzyme	Reference	Principal of Assay
**MRC Complex I**	[32]	NADH is oxidised complex I. Electrons are then transferred to CoQ_1_ (analogue of CoQ10), which is reduced to ubiquinol. Complex I activity is then measured by the rotenone-sensitive rate of NADH oxidation at 340 nm.
**MRC Complex II**	[32]	Succinate is oxidised by complex II and the resulting electrons are transferred from this enzyme via CoQ_1_ to DTNB (5,5′-dithio-bis-(2-nitrobenzoic acid)). Complex II activity is then measured by the succinate-dependent thenoyltrifluoroacetone (specific complex II inhibitor) reduction of DTNB at 600 nm.
**MRC Complex III**	[32]	Decylubiquinol (analogue of CoQ10) donates electrons to complex III, which then reduces cytochrome c (Cyt C). Complex III activity is then measured by the antimycin A (specific complex III inhibitor)-sensitive reduction of Cyt C at 550 nm.
**MRC Complex IV**	[32]	The potassium cyanide-sensitive oxidation of reduced Cyt. C by complex IV is measured at 550 nm. The activity is highly dependent on Cyt. C concentration. Consequently, activity is expressed as a pseudo-first-order rate constant.
**MRC Complex I–III**	[33]	NADH is oxidised by complex I. The electrons are then transferred to complex III by CoQ10, which then reduces Cyt C. The activity of complex I–III is measured by NADH-dependent, antimycin A-sensitive reduction of Cyt C at 550 nm.
**MRC Complex II–III**	[33]	Succinate is oxidised by complex II. Electrons are then transferred from complex II by CoQ10, which then reduces Cyt C. Complex II/III activity is measured by the succinate-dependent, antimycin A-sensitive reduction of Cyt C at 550 nm.
**Citrate synthase (CS)**	[32]	CS catalyses the condensation of oxaloacetate and acetyl-CoA to form citrate and free coenzyme A (CoA-SH). CoA-SH reacts with the compound DTNB to form thionitrobenzoate (TNB), which absorbs at 412 nm. The activity of CS is proportional to the amount of TNB formed.

## Data Availability

This is a review article and all the informed presented is available in the cited references.
